# 3D‐Printed Hydrogels from Recycled Cellulose for Biomedical Applications

**DOI:** 10.1002/cssc.202501734

**Published:** 2025-12-29

**Authors:** Sara Yousefshahi, Eric Pohl, Timo Sehn, Marcel Jungbluth, Birgit Huber, Christopher O. Klein, Sabine Beuermann, Michael A. R. Meier, Ute Schepers, Christian W. Schmitt, Patrick Théato

**Affiliations:** ^1^ Karlsruhe Institute of Technology Soft Matter Synthesis Laboratory Institute for Biological Interfaces III (IBG‐3) Eggenstein‐Leopoldshafen Germany; ^2^ Karlsruhe Institute of Technology Institute for Functional Interfaces ‐ Chemical Biology (IFG‐CB) Eggenstein‐Leopoldshafen Germany; ^3^ Karlsruhe Institute of Technology Institute of Organic Chemistry (IOC) and Institute of Biological and Chemical Systems – Functional Molecular Systems (IBCS‐FMS) Karlsruhe Germany; ^4^ Clausthal University of Technology Institute for Technical Chemistry Clausthal‐Zellerfeld Germany; ^5^ Karlsruhe Institute of Technology Institute for Chemical Technology and Polymer Chemistry (ITCP) Karlsruhe Germany

**Keywords:** cellulose, digital light processing (DLP) printing, hydrogel, recycling, wound healing

## Abstract

Growing environmental awareness has led to a shift in focus toward green chemistry and the development of more sustainable materials. Cellulose is one of the most abundant renewable polymers, providing stability and flexibility in plant cell walls. Because of these properties, it has often been used as a base material for textiles, which can be recycled and the cellulose recovered, making it a promising candidate for environmentally friendlier polymer synthesis. Herein, we show a sustainable method for recycling and modifying cellulose to facilitate photochemical crosslinking to attain biocompatible hydrogels under mild reaction conditions, which can thus also be used for the fabrication of complex 3D structures via digital light processing (DLP). This approach presents an excellent technique for the fabrication of customized cell scaffolds for biomedical applications, such as the use as a wound dressing to treat chronic wounds.

## Graphical abstract

Cellulose recovered from textile waste was transformed into photo‐crosslinkable hydrogels and processed by light‐induced 3D‐printing. The resulting elastic, shape‐stable structures demonstrate high printing fidelity and highlight the potential of recycled textiles as a more sustainable source for hydrogel‐based wound patch materials. Created in BioRender. Yousefshahi,S. (2025) https://BioRender.com/0omwu4l.

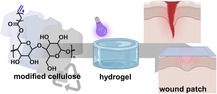

## 1 Introduction

The treatment of damaged skin remains a persisting challenge in the field of medicine. In the context of wound treatment, prompt treatment is important to prevent wound infection and the development of chronic wounds, which can have a significant impact on patient health. However, existing treatment options for scarless wound regeneration are limited. In recent years, several studies have explored the potential role of hydrogels in wound repair [1]. Currently, hydrogels for wound healing are primarily made from collagen or gelatin, derived from animal extracellular matrices, or from synthetic polymers like polyglycolic acid or polylactic acid in combination with water soluble crosslinkers [2]. However, many presently used wound dressing materials suffer from limitations such as poor antimicrobial efficacy, inadequate mechanical properties, and insufficient moisture retention, factors critical for effective wound healing [3]. At the same time, growing concerns about climate change and environmental impact have increased the demand for more sustainable processes and materials derived from renewable feedstocks. This has driven interest in bio‐based hydrogels that offer not only biocompatibility and biodegradability, but also improved functional performance and environmental compatibility [4, 5]. Polysaccharides, particularly cellulose as one of the most abundant organic polymers on earth, have shown considerable potential as raw materials for the previously mentioned purposes. Due to its biodegradability, renewability, and mechanical strength, cellulose finds application in food packaging, textiles, and, more recently, advanced manufacturing technologies such as 3D‐printing [4, 6]. Even tough cellulose shows excellent materials properties, direct 3D‐printing of cellulose faces challenges like thermal decomposition, as unmodified cellulose is not thermoplastic, thus requiring complex processing steps to obtain the desired materials, such as derivatization or dissolution in costly and potentially harmful solvents such as ionic liquids (IL) [7]. Generally, achieving suitable flow and solidification properties during extrusion printing is difficult and requires precise control over the material's rheological properties. Additionally, while cellulose is a renewable and more environmentally friendly material, the solvents and chemicals used in its chemical modification are mostly fossil‐based and may pose environmental and health risks. This is particularly the case in photoinduced 3D‐printing, such as digital light processing (DLP), where cellulose is often functionalized with methacrylate moieties [7]. Yet, the functionalization of cellulose is a crucial step to overcome solubility and compatibility issues with, for example, biological environments. Industrial cellulose modification is mainly conducted in a heterogeneous fashion. A prominent example is the acetic acid process for the synthesis of cellulose acetate (CA), where acetic anhydride, acetic acid, and sulfuric acid are employed. Unfortunately, heterogenous modification processes, such as the acetic acid process, suffer from drawbacks like a limited control of the degree of substitution (DS), an uneven distribution of substituents, and backbone degradation due to typically harsh reaction conditions. More precisely, to achieve the targeted DS of CA requires two steps, i.e., peracetylation and subsequent defunctionalization. Additionally, partial degradation is unavoidable due to these harsh reaction conditions, which lowers the molecular weight (MW) of the respective polymers and limits their final materials properties. On the contrary, homogenous cellulose modification, which can be performed in polar salt systems, such as IL or switchable solvent systems, allows for the direct adjustment of the DS and minimizes backbone degradation and is thus superior compared to their heterogeneous counterparts [6]. Particularly, the switchable solvent system approach introduced by Xie et al. and Jérome et al. in 2013 evolved as a greener and more effective alternative for cellulose modification compared to previous published studies. Especially, the easier recyclability of the components employed during the derivatization via distillation and the stoichiometric amount of transesterification agent are major benefits [8, 9].

Herein, we employ a more environmentally friendly alternative to their introduced method, involving the modification of cellulose with functional esters to increase its solubility via a switchable solvent system, which enables the introduction of terminal C—C double bonds onto the cellulose backbone, which can serve as reactive sites for further chemical functionalization [6]. By incorporating these alkene groups, the modified cellulose also becomes suitable for subsequent network formation via efficient thiol‐ene chemistry, allowing the formation of covalently crosslinked cellulose‐based hydrogels when reacted with di‐ and polythiols [10]. The crosslinking process can be initiated using light, which can be precisely controlled in both spatial and temporal terms, enabling the design of the morphology and structure of the materials. This procedure allows the creation of complex 3D structures via 3D‐printing (e.g., DLP), which can serve as tissue‐specific scaffolds for wound dressings, offering a sustainable and innovative solution to current medical challenges [11, 12, 13].

**FIGURE 1:**
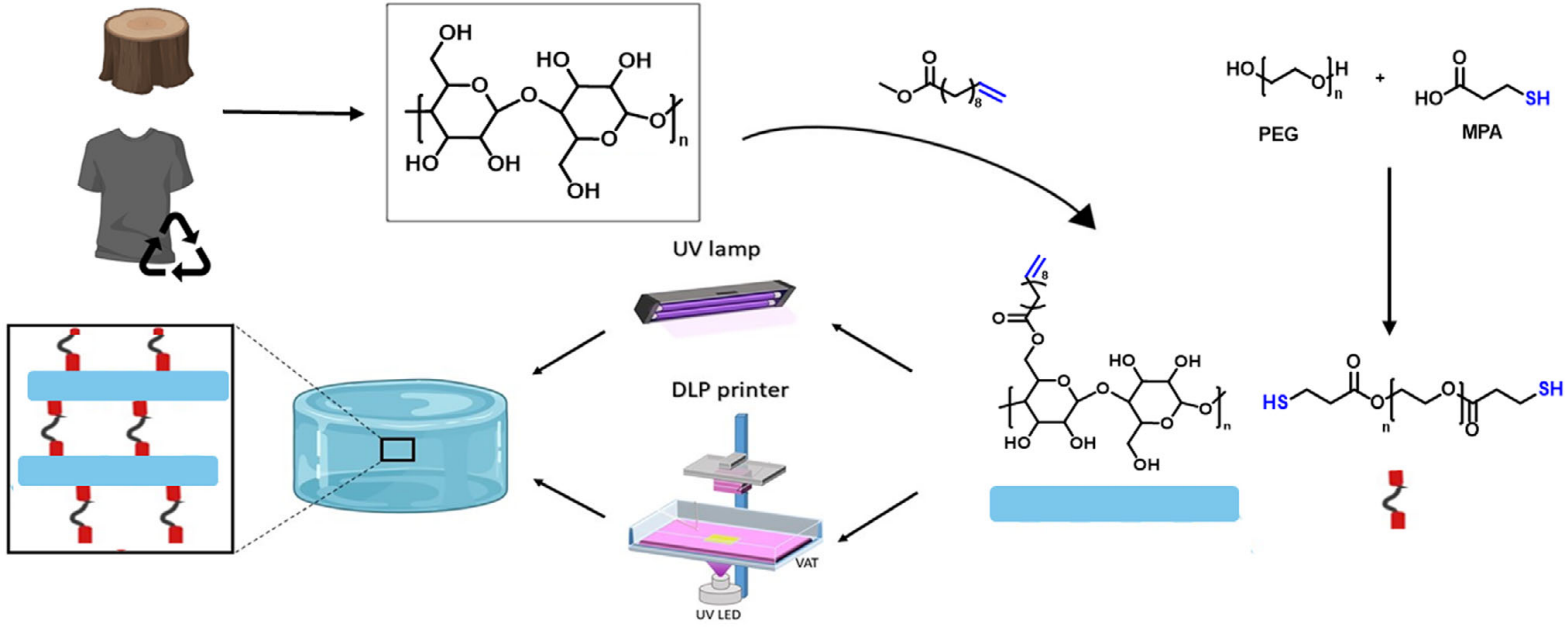
Overview of the steps for preparing cellulose‐based hydrogels. Cellulose from plant fibers or recycled textiles is functionalized with methyl 10‐undecenoate and crosslinked with a polyethylene glycol (PEG)‐dithiol via the photo‐induced thiol‐ene reaction with an ultraviolet (UV) lamp or a DLP printer to form hydrogels.

Besides using commercial cellulose, we further investigated the possibility of processing recycled cellulose from waste textiles. Since the textile industry is one of the largest contributors to global waste, with over 92 million tons of textile waste annually, it is crucial to find ways to recycle textiles into valuable materials. Due to the insolubility of cellulose, extracting and recycling cellulose from textiles presents significant challenges. The process involves not only collecting and cleaning waste textiles but also separating cellulose from other components such as additives, dyes, and synthetic polymers. Achieving a clean separation is crucial for advanced manufacturing processes, as it ensures consistent processability and the desired properties of the final product. Therefore, the potential of cellulose extracted from polycotton blends was investigated once again using the switchable solvent system. [14, 15]; Despite these advances, sustainable strategies for developing cellulose‐based hydrogels that combine environmental compatibility with tailored functionality for biomedical applications remain underexplored. In this work, as illustrated in Figure 1 this challenge, is addressed by employing a switchable solvent system to introduce alkene functionalities onto cellulose via esterification employing renewable methyl 10‐undecenoate. This approach enables efficient crosslinking via thiol‐ene chemistry compatible with 3D‐printing, resulting in bio‐based hydrogels with promising properties for wound healing applications. To further increase sustainability, the approach was also successfully applied to recycled cellulose derived from polycotton waste, demonstrating the potential for circular material use in biomedical hydrogel design.

## 2 Results and Discussion

### 2.1 Functionalization of Cellulose

A suitable chemical derivatization is highly desirable for the synthesis of hydrogels from cellulose. However, as cellulose is insoluble in water and most organic solvents, a suitable solvent is required. Here, the aforementioned DMSO/TBD/CO_2_ switchable solvent system was applied and methyl 10‐undecenoate was used for transesterification to introduce terminal C=C double bonds, enabling a subsequent crosslinking via thiol‐ene chemistry [6, 16, 17, 18, 19]. The use of methyl 10‐undecenoate is motivated by sustainability considerations because it is derived from castor oil, a commercially available and renewable resource [20].

**FIGURE 2:**
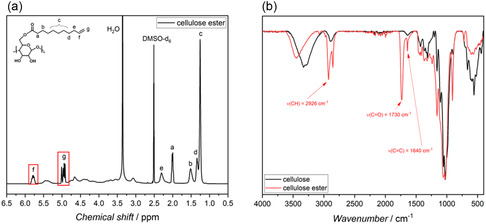
Structural characterization of cellulose undecenoate via ^1^H NMR and FTIR spectroscopy. (a) ^1^H NMR of MCC after functionalization dissolved in DMSO‐d_6_. (b) ATR‐FTIR of MCC after functionalization compared to unfunctionalized cellulose.

For this, microcrystalline cellulose (MCC) was dissolved in the DMSO/TBD/CO_2_ switchable solvent system and modified with methyl 10‐undecenoate. To confirm the successful modification of cellulose with methyl 10‐undecenoate, both ^1^H nuclear magnetic resonance spectroscopy (NMR) and attenuated total reflectance Fourier transform infrared spectroscopy (ATR‐FTIR) spectra were recorded. The ^1^H NMR spectrum (Figure 2a) shows distinct signals of the double bond introduced through esterification. In particular, the peaks at 5.85–5.72 ppm (*f*) and 5.03–4.89 ppm (*g*) correspond to the vinylic protons of the terminal C=C double bond, which indicates that the methyl 10‐undecenoate was successfully attached to the cellulose backbone.

The FTIR spectrum (Figure 2b) further supported these findings. A strong signal at 1730 cm^−1^ confirmed the presence of the ester carbonyl group (C=O), while an additional peak at 1640 cm^−1^ is assigned to the C=C bond of the alkene. Compared to unmodified cellulose, the broad hydroxyl band at 3432 cm^−1^ is reduced, suggesting that hydroxyl groups were successfully substituted. These results confirm that methyl 10‐undecenoate was covalently bound to the cellulose structure. Based on the integration of the relevant NMR signals (Figure S1, Supporting Information), a DS of 1.05 was determined, indicating that, on average, one hydroxyl group per glucose unit was functionalized. To accurately quantify the DS, the cellulose derivative was analyzed by ^31^P NMR after phosphitylation in a CDCl_3_/pyridine mixture as a solvent system, since measurements in DMSO‐d_6_ led to overlap of the double bonds with the backbone signals of cellulose, making reliable integration and thus accurate determination of the DS impossible.

### 2.2 Extraction of Cellulose From Textile Waste

Polycotton is a fabric blend made from polyester (mostly polyethylene terephthalate, PET) and cotton, combining the durability and wrinkle resistance of polyesters with the breathability and softness of cotton and is therefore commonly used in clothing and bed linens. Yet, the blended fabric is hard to separate using mechanical recycling methods [21, 22, 23]. Thus, we herein present a chemical separation process. For this, polycotton cutting remnants containing 50 wt% PET and 50 wt% cotton were separated into their individual components using a DMSO/DBU/CO_2_ switchable solvent system. This system enables the selective dissolution of cellulose under mild conditions by reversibly forming carbonate anions in DMSO, thus breaking hydrogen bonds in cellulose without affecting the polyester component [15]. In our approach, three experiments (#1, #2, and #3) were carried out in a two‐step extraction process at 40°C for 6 h each, allowing for efficient recovery of the cellulose fraction while preserving the integrity of the polyester. The remaining textiles and extracted cellulose were analyzed using thermogravimetric analysis (TGA). Since cellulose and PET decompose at temperatures around 350 and 430°C, respectively [24, 25, 26, 27], the composition of the samples was estimated from the TGA measurements. The TGA curves of the residual textile #1 and #3 do not show an indication of residual cellulose. In sample #2, a small amount of cellulose remained in the textile residue, likely due to residual moisture in DMSO, which interfered with the solvent activation and hindered full dissolution. The results are summarized in Table 1. The observed loss of cellulose during the process was due to the several washing steps and led to cellulose recovery between 82% and 93%. Samples #1 and #3, provide the cellulose for the subsequent modification. Further analysis by NMR was not possible, since PET is inert to most commonly used NMR‐solvents.

**TABLE 1:** Masses of virgin polycotton, thereof extracted cellulose and estimated composition of the remaining textile according to TGA analysis by calculating the ratio of the individual mass loss to the total mass loss.

Extraction	Mass of polycotton, g	Mass of extracted cellulose, g	Cellulose recovered, %	Composition of remaining textile
#1	2.6373	1.2300	93	100% PET
#2	2.6380	1.0760	82	97% PET /3% Cellulose
#3	2.6437	1.1475	87	100% PET

TGA analyses of the extracted cellulose showed one large weight loss step at 338°C for extraction #1 and #2, corresponding to the decomposition temperature (*T*_d_) as determined by the inflection point of the weight loss curve. The cellulose from the extraction #3 shows a second degradation step at 278°C, most probable due to remaining traces of DBU (Figure S2, Supporting Information). A degradation step for PET was not observed for all TGA analyses of the extracted cellulose. Due to the dissolution and reprecipitation of the extracted cellulose, the peak degradation temperature of 338°C was lowered slightly compared to cellulose in the virgin polycotton. This might be caused by the changes in crystallinity, which has an influence on the thermal degradation, as described in the literature [28]. The small fraction of DBU in sample #3 was not hindering the following dissolution for functionalization of the cellulose, since DBU was used as part of the solvent mixture in the subsequent functionalization of cellulose.

**FIGURE 3:**
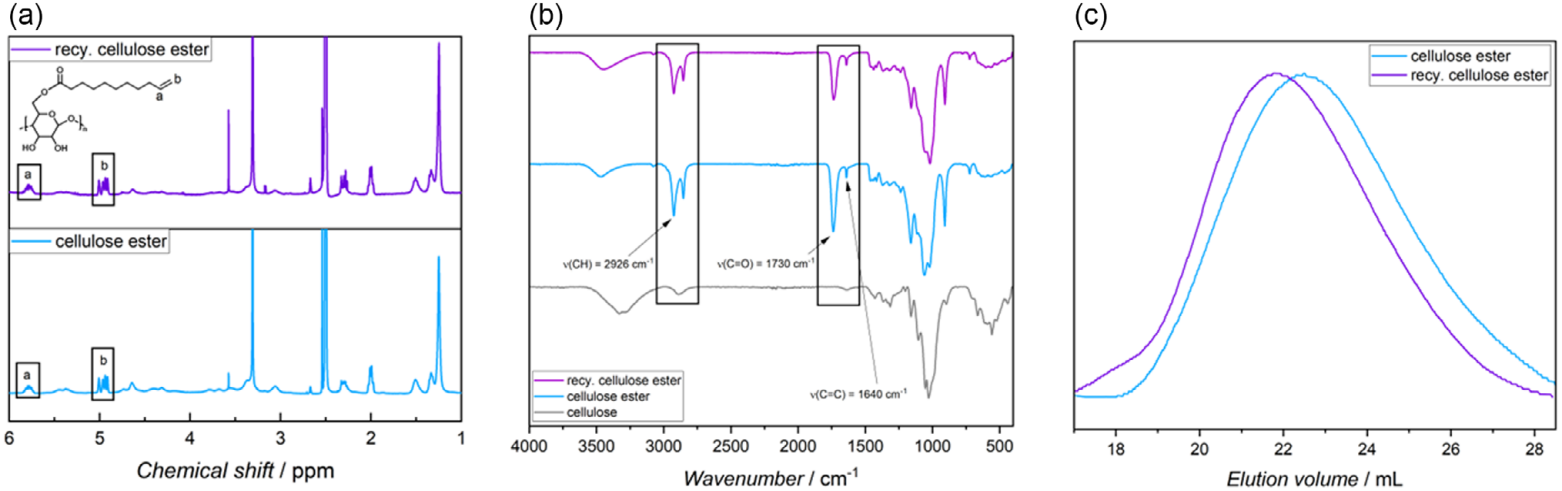
Analysis of functionalized MCC and differences to recycled cellulose. (a) ^1^H NMR of MCC and recycled cellulose after functionalization in DMSO‐d_6_. (b) ATR‐FTIR of MCC and recycled cellulose after functionalization. (c) SEC measurements of MCC and recycled cellulose after functionalization in DMAc. The M_*n*_ evaluation was done by calibration with pullulan standards.

The first attempt to functionalize the recycled cellulose combined the extraction and modification of cellulose into one process. According to the literature, extractions of 4 wt% polycotton and 10 wt% DBU in 30 mL of DMSO were carried out for 6 h [15]. The obtained cellulose solution can be used for modification reactions, as was demonstrated for various transesterifications with cellulose [6, 16, 17, 18, 19]. Herein, the dissolved cellulose was modified, with using 6 eq. of methyl 10‐undecenoate per anhydroglucose unit (AGU) of the cellulose content in the polycotton. Since the cellulose contained in the polycotton is not completely dissolved in a single extraction, the molar ratio of methyl 10‐undecenoate to cellulose is expected to be significantly higher. The modified cellulose was analyzed using ATR‐FTIR spectrometry, as shown in Figure S3 (Supporting Information) new IR bands at 1734 and 1640 cm^−1^ were observed, which are characteristic to the C=O bond and the C=C double bond. These bands were indicative of a successful modification of cellulose with methyl 10‐undecenoate, in agreement with reports in the literature [15]. Yet, the modified cellulose was poorly soluble in DMSO, dimethylacetamide (DMAc), or dimethylformamide (DMF), most likely due to a low DS. Nevertheless, this provides proof of concept that the process of cellulose dissolution from polycotton can be combined with a subsequent modification of cellulose; however, the optimization of this process is subject to future research. Therefore, the functionalization of the recycled cellulose was carried out in a separate process, as described for the MCC. The successful functionalization of the recycled cellulose was also confirmed by ^1^H NMR and ATR‐FTIR spectroscopy, where in the ^1^H NMR spectrum (Figure 3a), the appearance of signals in the range of (a) 5.85–5.72 ppm and (b) 5.03–4.89 ppm, corresponding to the vinylic protons of the terminal double bond, indicates the incorporation of methyl 10‐undecenoate into the cellulose backbone via esterification. Complementary evidence was provided by the ATR‐FTIR spectrum (Figure 3b), which showed a distinct carbonyl stretching vibration at 1730 cm^−1^, characteristic of ester functionalities, and a peak at 1640 cm^−1^ attributed to the C=C bond. Moreover, a decrease in the intensity of the broad O–H stretching band at 3432 cm^−1^ compared to the unmodified sample suggested successful substitution of hydroxyl groups. The DS was only determined for the MCC derivative, as the recycled cellulose could not be evaluated due to limited solubility under the conditions required for ^31^P NMR analysis. The successful functionalization of both cellulose types confirmed the applicability of the switchable solvent system to materials that are soluble in DMSO.

Interestingly, both cellulose types differed in their MW, as shown by the size exclusion chromatography (SEC) measurements in Figure 3c). The results show that the M_*n*_ of the recycled cellulose is 180 kg/mol, about 1.8 times greater than that of the MCC (100 kg/mol), with a similar dispersity of 7.4. The discrepancy can be attributed to different origins of the cellulose fibers or its processing, since the recycled cellulose was recovered from polycotton blends, making it cotton‐based, while the purchased cellulose (in this case MCC) is derived from plant fibers [29].

### 2.3 Hydrogel Formation

The cellulose‐based hydrogels were prepared using PEG‐dithiols as crosslinkers via the thiol‐ene reaction, thereby creating a covalent bond between the two components. The successful thiol‐functionalization of PEGs was confirmed by ^1^H NMR and ATR‐FTIR analysis, showing the expected chemical shifts at 1.62–1.58 ppm for the mercaptane and IR signals at 1731 cm^−1^ for the ester groups, respectively (see Figures S7 and S8, Supporting Information). Due to its biocompatibility and hydrophilicity—even though not from renewable resources—PEG is an optimal choice for preparing hydrogels, offering a cost‐effective and straightforward functionalization process [30]. Different M_*n*_ of PEG were investigated to be able to vary the different of the hydrogels, such as swelling and mechanical stability. An overview of all hydrogel formulations, including the cellulose type and PEG‐dithiol M_*n*_, is provided in Table 2. The crosslinking was photochemically conducted using lithium‐phenyl‐2,4,6‐trimethylbenzoylphosphinat (LAP) as photoinitiator, because it decomposes at 405 nm, rendering it potentially biocompatible [31].

**TABLE 2:** Overview of the hydrogel formulations based on cellulose source and PEG‐dithiol crosslinker.

Sample	Cellulose type	M_*n*_ of PEG‐dithiol, g/mol
MCC‐300	MCC	300
MCC‐600	MCC	600
MCC‐2000	MCC	2000
MCC‐6000	MCC	6000
Recy‐300	Recycled cellulose	300
Recy‐600	Recycled cellulose	600
Recy‐2000	Recycled cellulose	2000
Recy‐6000	Recycled cellulose	6000

**FIGURE 4:**
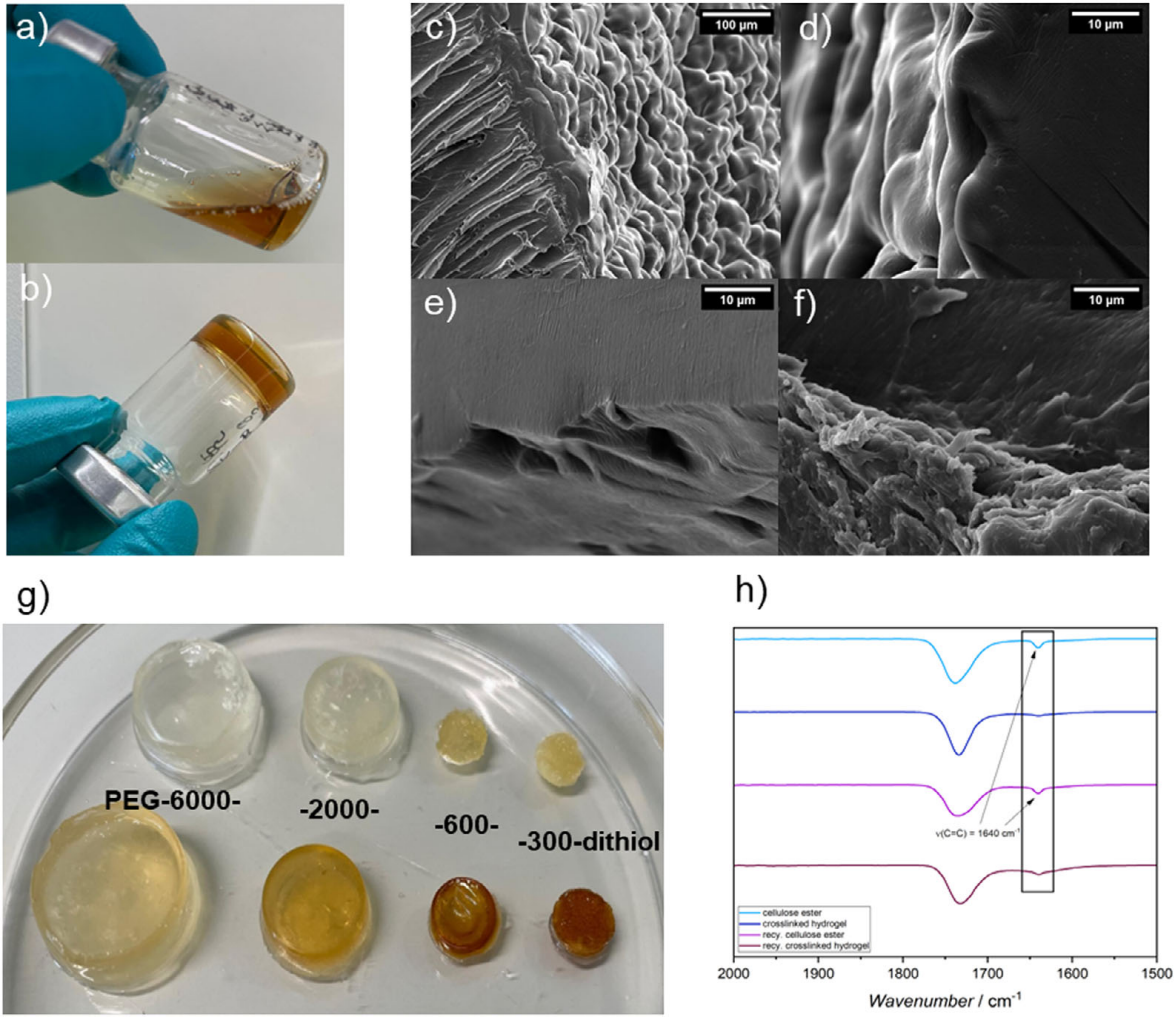
Synthesis of cellulose‐based hydrogels. (a) 3 wt% cellulose solution dissolved in DMSO with 1:1.5 crosslinker ratio and 0.5 mg/mL LAP in DMSO before and (b) after irradiation with light at 405 nm. (c–f) SEM measurements of freeze‐dried hydrogels with (c) PEG‐300‐, (d) ‐600‐, (e) ‐2000‐, and (f) ‐6000‐dithiol. Samples were sputter‐coated with platin and imaged at an accelerating voltage of 10 kV and at a magnification of 5kx. (g) Hydrogels in the swollen state with PEG‐300‐, ‐600‐, ‐2000‐, and ‐6000‐dithiol with recycled (top) and MCC‐based (bottom) cellulose. (h) ATR‐FTIR spectrum of modified commercial and recycled cellulose and of the respective crosslinked hydrogels.

As shown in Figure 4a,b, the cellulose–PEG solution containing LAP transitioned from a liquid to a solid state upon light exposure, indicating successful gel formation. It can therefore be assumed that the network formation via thiol‐ene coupling was successful, which is supported by the ATR‐FTIR spectrum (Figure 4h), which demonstrated the decrease of the C=C double bond signal after crosslinking. Yet, small amounts of C=C double bonds remained unreacted, which permits further functionalization of the hydrogel. In Figure 4g), hydrogels were produced using recycled modified cellulose (top row) and commercial modified cellulose (bottom row). The crosslinkers vary in structure from PEG‐6000‐dithiol (on the left) to PEG‐300‐dithiol (on the right). It is evident that the hydrogel formation was successful with the recycled modified cellulose and all the different crosslinkers. The gelation time decreased with increasing LAP concentration and shorter PEG chain length, indicating faster network formation under higher radical concentration. Conversely, longer PEG chains led to slightly delayed gelation due to reduced chain mobility. To evaluate the physical properties of the resulting hydrogels, their swelling behavior in water was investigated. M_*n*_ of the crosslinker is directly correlated with swelling of the respective networks in water. This result can be attributed to the larger mesh size of the hydrogel, which allowed for increased water absorption. This behavior was confirmed by swelling tests (see Figure S10, Supporting Information). The water absorption of the hydrogels was quantified at designated time points (0.25, 0.5, 1, 2, 3, 4, 5 and 12 h) over a 12 h period in a triple determination. The degree of water absorption was calculated based on the weight of the hydrogels and expressed as a percentage. Hydrogels prepared with higher MW PEGs (PEG 2000 and PEG 6000) exhibited significantly higher equilibrium swelling ratios compared to those crosslinked with shorter PEGs (PEG 300 and PEG 600), indicating a lower crosslinking density and correspondingly larger mesh sizes. Specifically, MCC‐based hydrogels reached maximum swelling ratios of 39% ± 0.4%, 40% ± 0.1%, 78% ± 0.3%, and 87% ± 0.5% for PEG 300, 600, 2000, and 6000, respectively. A similar trend was observed for the hydrogels derived from recycled cellulose, with values of 32% ± 1.7%, 42% ± 0.4%, 78% ± 0.3%, and 82% ± 1.2%, respectively.

All formulations showed rapid initial swelling, reaching equilibrium within approximately 1–2 h, demonstrating fast water uptake kinetics. Notably, the recycled cellulose‐based hydrogels displayed swelling kinetics and equilibrium values comparable to those based on MCC, supporting the suitability of recycled cellulose as a sustainable and functionally equivalent alternative.

Another factor influencing swelling is the DS of the cellulose backbone with alkene groups, which directly determines the number of available reactive sites for thiol‐ene crosslinking. A lower DS can lead to reduced network formation and a lower crosslinking density, thereby increasing mesh size and enhancing swelling. In this context, the comparable swelling behavior between MCC and recycled cellulose suggests that the functionalization was sufficiently efficient in both cases to enable effective crosslinking.

The water absorption or water loss of the fully swollen hydrogels was confirmed using TGA (Figure S11 in the Supporting Information). Two weight loss stages are evident in the diagrams, occurring at approximately 100°C and between 400 and 600°C. It can be postulated that the initial reduction in mass is indicative of the loss of water from the hydrogels. The water loss observed in these experiments is consistent with the results of the previously described swelling tests. These results deviate by only 2%–6% from those of the swelling tests. In the second stage of weight loss, the degradation of the network structure of the hydrogels can be postulated, which was confirmed by the TGA measurement of the dried gels (Figure S12 in the Supporting Information). All hydrogel samples showed degradation in the range of 400–600°C.

Subsequently, scanning electron microscopy (SEM) images were recorded to gain a more detailed understanding of the structural characteristics of the hydrogels. Figure 4c–f depict crosslinked hydrogels, which were processed via freeze‐drying. The images demonstrate that the hydrogel exhibits a porous structure and lacks a smooth surface. This phenomenon was attributed to the formation of a network with meshes in the hydrogel, which allows for the trapping of water molecules. This typical structure enabled the hydrogels to swell without compromising their structural integrity, as the defined mesh size allows for controlled expansion of the polymer network [32].

**FIGURE 5:**
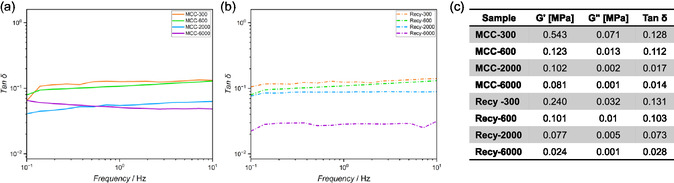
Oscillatory shear of swollen hydrogels produced from commercial and recycled cellulose with PEG‐300‐, ‐600‐, ‐2000‐, and ‐6000‐dithiols. (a) Graphic representation of rheology measurements of swollen hydrogels made from MCC with PEG‐300‐, ‐600‐, ‐2000‐, and ‐6000‐dithiols. (b) Graphic representation of rheological measurements of swollen hydrogels made from recycled cellulose with PEG‐300‐, ‐600‐, ‐2000‐, and ‐6000‐dithiols. (c) Storage modulus (G′), loss modulus (G″), and loss factor (tan *δ*) of different hydrogel formulations.

The elasticity of hydrogels is closely related to their mesh size, as a smaller mesh size, resulting from a higher crosslinking density, typically leads to increased stiffness and more elastic behavior. To evaluate the viscoelastic properties of the hydrogels, frequency sweep measurements were performed and the loss factor tan δ, defined as the ratio of the loss modulus (*G″*) to the storage modulus (*G′*), was determined. *G′* represents the energy stored during deformation and reflects hydrogel stiffness, while *G″* quantifies the energy dissipated during shearing and is associated with viscous flow. A tan δ value below 1 (*G′*  > *G″*) indicates a dominantly elastic response, whereas a value above 1 reflects viscous behavior. All tested hydrogels displayed tan δ values below 1 across the measured frequency range (see Figure 5), indicating that elastic contributions dominate [33].

Hydrogels crosslinked with PEG‐300 exhibited higher tan *δ* values, indicating a relatively more pronounced viscous contribution compared to PEG‐6000‐based hydrogels. This is consistent with the expected trend, as hydrogels prepared with higher MW crosslinkers showed lower crosslinking densities and larger mesh sizes, which translate to increased pliability and lower stiffness. The differences in G′ and tan *δ* values correlate well with the hydrogel microstructure observed by SEM. Samples with higher storage moduli (e.g., MCC‐300) exhibited denser and more homogeneous pore structures, whereas recycled cellulose hydrogels with slightly lower G′ values showed more open and irregular porosity, suggesting reduced crosslinking density.

The hydrogels prepared from recycled cellulose exhibited comparable tan δ values to those of their commercial cellulose counterparts. These values range from 0.01 to 0.2, indicating highly elastic networks with storage moduli that exceed loss moduli by about a factor of 10 (see Figure 5a). Quantitatively, G′ of recycled cellulose hydrogels was on average 42% lower than that of MCC‐derived samples, while maintaining similarly low tan *δ* values (<0.15), confirming a predominantly elastic network structure. Despite lower absolute moduli, the viscoelastic performance and qualitative network behavior remained comparable between recycled and commercial cellulose hydrogels. These results suggest that recycled cellulose as a more sustainable alternative is suitable for producing hydrogels with comparable mechanical integrity and viscoelastic performance. Thus, further tests of hydrogel application possibilities were performed with recovered cellulose samples.

### 2.4 Wound Patches

**FIGURE 6:**
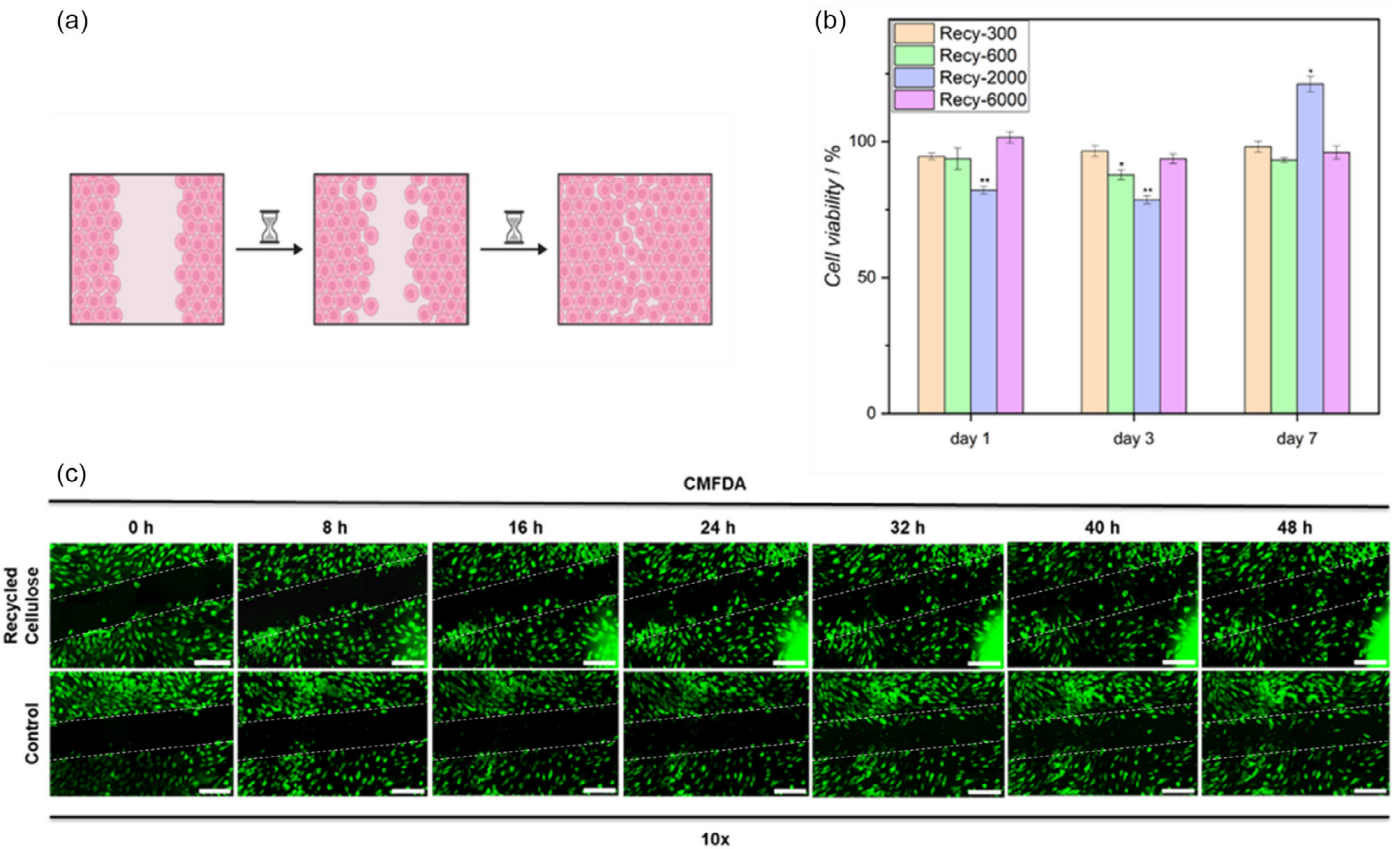
(a) Schematic representation of the application of hydrogels for wound closure. Created in BioRender. Yousefshahi, S. (2025). https://BioRender.com/encdnq3. (b) Determination of cell viability of recycled cellulose hydrogels after 1, 3, and 7 days of leaching by analyzing formazan conversion. Differences were considered statistically significant at a level of *p* < 0.05. Asterisks indicate significant deviations from the control medium (*p < 0.05; **p < 0.01; ***p < 0.001). (c) Fluorescence microscopy of CMFDA‐labeled NHDF after 0, 8, 16, 24, 32, 40, and 48 h treated with recycled and purchased cellulose‐based hydrogels. NHDF without hydrogel treatment served as a control. Scalebar equals 1 mm.

In the following, the prepared hydrogels were evaluated with a particular emphasis on their potential as wound dressings by assessing key properties essential for effective wound healing. Specifically, their cytocompatibility and ability to promote wound closure were investigated to determine their suitability as wound patches. The 3‐(4,5‐dimethylthiazol‐2‐yl)−2,5‐diphenyltetrazoliumbromid (MTT) assay was employed as a viability test to evaluate the material's impact on cellular health. MTT, a water‐soluble tetrazolium salt, is metabolized in living cells by succinate dehydrogenase, a mitochondrial enzyme, to formazan, an insoluble compound. This transformation results in a color change from yellow to blue‐purple, which can be quantitatively assessed using a photometer at a wavelength of 595 nm. This method provides a quantitative measurement of cell viability [34]. In our experiments, hydrogels based on recycled cellulose crosslinked with aforementioned PEG‐dithiols were leached in cell culture medium, which was collected on days 1, 3, and 7 to assess whether any cytotoxic substances leached from the hydrogels over time. The collected media were then applied to cultured human dermal fibroblasts (NHDF), and cell viability was measured using an MTT assay to evaluate the hydrogel's potential biocompatibility. As illustrated in Figure 6b, the viability of the hydrogels ranged from 79% to 121%, with a standard deviation of less than 5% in a triplicate determination. As light reduction in metabolic activity was seen, although cell viability remained above 90% for most samples. In general, prolonged leaching times led to a slight increase in cell viability, likely due to the gradual degradation or dilution of cytotoxic side products released from the hydrogels. To further substantiate these observations, statistical analysis using a two‐tailed Student's t‐test was performed to compare each sample with the control. While most samples showed no statistically significant differences (*p* > 0.05), the Recy‐2000 hydrogel exhibited a significant increase in cell viability compared to the control on day 7 (*p* < 0.05). Notably, hydrogels showed consistent and stable viability values, independent of the crosslinker.

The most significant improvement in metabolic activity was observed in hydrogels composed of recycled cellulose crosslinked with PEG‐2000. This statistically confirmed enhancement may indicate a potential bioactive or stimulatory effect, giving rise to further exploration of this material as a wound dressing.

To prove the impact of hydrogels on wound healing, a scratch assay was performed. This is an in vitro procedure in which a cell monolayer is mechanically injured to analyze cellular migration and wound healing capacity over time [35]. NHDF were seeded in an 8‐well ibiTreat slide and scratched to produce a wound in the cell layer. Eventually, the wound was covered with the crosslinked hydrogel 7d after lavage. Microscopic images of the hydrogel‐treated cells and the untreated control were obtained at several time points over a period of 48 h. Quantitative analysis of wound closure was performed by applying cell masks to determine confluency changes over time. As illustrated in Figure 6c, hydrogels based on recycled cellulose slightly accelerated wound closure, with the confluency increasing by approximately 11.3% compared to 6.6% in the untreated control, indicating faster cell remigration into the scratched area. No phenotypical abnormalities were observed in these samples.

In conclusion, recycled cellulose‐based hydrogels, particularly in combination with PEG‐2000 demonstrate favorable effects on cell viability, migration, and proliferation, highlighting their potential as promising candidates for wound dressing applications.

### 2.5 3D‐Printing

**FIGURE 7:**
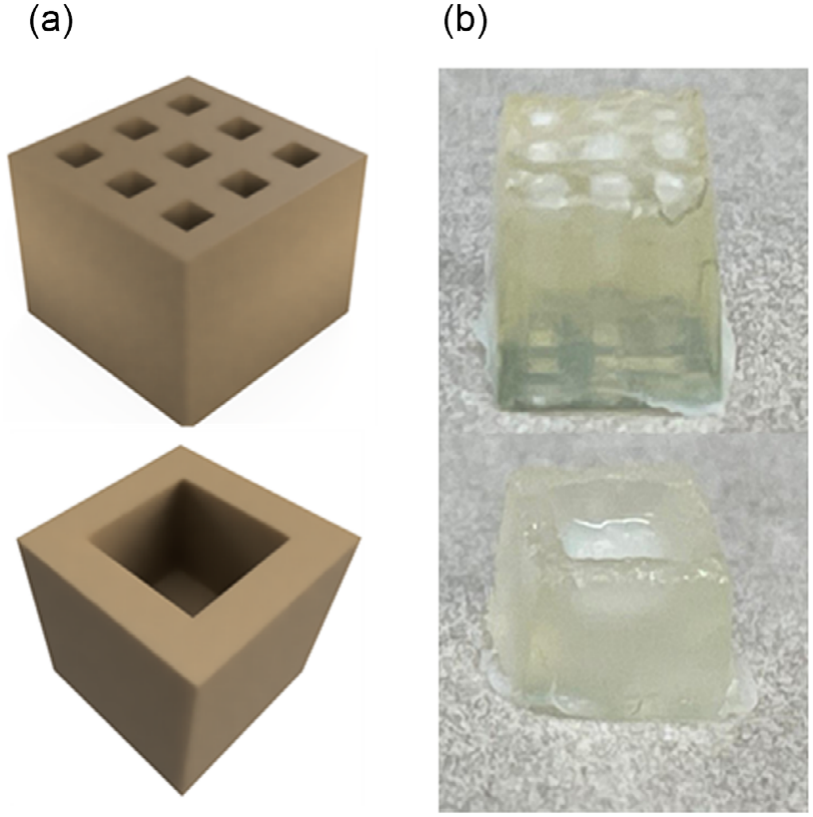
3D‐printing of cellulose‐based hydrogels. (a) CAD designed 6 × 6 × 6 mm cube with nine channels and hollow cube with a recess of 2 × 2 × 2 mm. (b) 3D‐printed cubes of recycled cellulose from a 3 wt% solution with 1:1.5 crosslinker ratio and 1 mg/mL LAP in DMSO.

The utilization of 3D‐printers for the processing of hydrogels enables the creation of targeted structures, for instance, for use as wound dressings, thereby facilitating the replication of patient‐specific structural arrangements. A photoinitiated crosslinking reaction enables the control of the reaction in terms of time and space through light irradiation, thus facilitating the creation of specific gels via DLP technique [11, 12, 13]. In initial printing tests, identical parameters and concentrations were employed as those utilized for gel formation described above. The solutions were prepared with a concentration of 3 wt% cellulose and a ratio of 1:1.5 of cellulose to crosslinker. Recycled cellulose was chosen to promote a more sustainable approach by utilizing waste‐derived materials as part of a Circular Economy. In addition, while PEG‐2000 was selected as a crosslinker, it offers a balanced combination of flexibility and mechanical stability, which are advantageous for hydrogel performance in wound dressing applications. As a photoinitiator, 1 mg/mL LAP and a printing speed ratio of 0.01 mm/s was used and the first gels were created from the CAD model (Figure 7a) in a printing time of 12 min (Figure 7b) with a lateral resolution of 10 µm and a z‐precision of 4 µm. Printing at an increased speed of 0.02 mm/s (6 min total) yielded constructs of comparable quality and demonstrated high reproducibility across multiple prints. After postcuring, the constructs retained their dimensional stability and mechanical integrity even after months of storage under aqueous conditions (see Figure S14, Supporting Information), confirming that both printing precision and long‐term structural robustness are suitable for potential biomedical applications. It can be concluded that cellulose‐based resins functionalized with terminal alkene groups can be spatially polymerized via thiol‐ene chemistry under light exposure, enabling precise control over the hydrogel's architecture. This allows for the direct fabrication of customizable 3D structures using light‐based techniques without the need for methacrylate modifications or toxic solvents. This approach overcomes key limitations of cellulose 3D‐printing by providing a resin system that combines biocompatibility, sustainability, and printability, paving the way for environmentally friendly and patient‐specific wound dressings.

## 3 Conclusion

The functionalization of cellulose was achieved via a homogeneous and more sustainable switchable solvent system, followed by crosslinking with PEG‐dithiols to form hydrogels. Importantly, the developed approach proved equally effective when using recycled cellulose obtained from polycotton, highlighting the potential for creating biomaterials from textile residues and contributing to a more sustainable material cycle. Moreover, hydrogels crosslinked with PEG‐2000 demonstrated particularly promising properties, combining favorable elasticity with good structural stability, making them especially suitable for 3D‐printing and wound dressing applications where both flexibility and mechanical integrity are essential. While the 3D printing of cellulose from recycled textiles holds significant promise, future efforts should aim to improve the scalability of the recycling process, ensure consistent cellulose quality, and optimize printing formulations. By addressing these challenges, the range of sustainable and high‐performance applications for cellulose‐based materials could be greatly expanded.

## 4 Experimental Section

### 4.1 Materials

Cellulose was purchased from Sigma–Aldrich and recycled cellulose was obtained from polycotton (50% polyester, 50% cotton) from Dibella BV. 1,5,7‐Triazabicyclo(4.4.0)dec‐5‐en (TBD, >98%, TCI), 3‐mercaptopropionic acid (MPA, >=99%, Sigma–Aldrich), aluminum oxide (Sigma–Aldrich), CO_2_ (4.5, Westfalengas), diazabicyclo [5.4.0]undec–7–en (DBU, >98%, TCI), diethyl ether (>99.7%, VWR), DMAc (>=99.8%, VWR), dimethyl sulfoxide (DMSO, Carl Roth), DMSO (TCI), DMSO (dry and stored over molecular sieve, Acros Organics), ethanol (>95%, Nordbrand Nordhausen GmbH), LAP (>=95%, Angene Chemical), methanol (>=99.8%, Thermo Fisher), methyl‐10‐undecenoate (>96%, TCI), *para*‐toluene sulfonic acid (*p*‐TSA, >=98.5%, Sigmal‐Aldrich), PEG (Sigmal‐Aldrich), toluene (99%, Thermo Scientific) were used without further purification. Deuterated solvents (DMSO‐d_6_ and CDCl_3_) were purchased from Eurisotop. For biomedical applications MTT, cellTracker 5‐chloromethylfluorescein diacetate (CMFDA), Dulbecco's Phosphate Buffered Saline (DPBS), Dulbecco's Modified Eagle Medium (DMEM), fetal bovine serum (FBS), penicillin‐streptomycin, L‐alanyl‐L‐glutamine, triton X‐100‐solution, and trypsin/EDTA were all purchased from Thermo Fisher. Normal human dermal fibroblasts (NHDF) were isolated from the dermis of adult skin and were provided by PromoCell.

### 4.2 Instrumentation

#### 4.2.1 NMR Spectroscopy

The Bruker Avance 400 (400 MHz) was used to record ^1^H NMR spectra for analysis of the products. The products were dissolved in NMR tubes with deuterated solvents. The classification of the peaks obtained was referenced to the solvent peak. The solvents used were CDCl_3_ (7.26 ppm) and DMSO‐*d*_6_ (2.50 ppm).

#### 4.2.2 ATR‐FTIR Spectroscopy

The Bruker Alpha II ATR‐IR spectrometer was used to analyze the products by measuring FTIR spectra in the range from 400 to 4,000 cm^−1^ at room temperature. 16 scans were conducted with a spectral resolution of 4 cm^−1^.

#### 4.2.3 SEC

SEC measurements were performed on a Agilent Technologies 1260 Infinity, comprising an autosampler, a GRAM 10 μm bead‐size guard column (8 × 50 mm, PSS) followed by three GRAM 10 μm columns (8 × 300 mm, subsequently 100, 3000, and 3000 Å pore size, PSS), and a differential refractive index (DRI) detector using DMAc with 0,316 g/L Lithiumbromid as the eluent at 60°C with a flow rate of 1 mL*min‐1. The SEC system was calibrated using Pullulan standards (PSS) ranging from 1030 to 708 000 g/mol.

#### 4.2.4 SEM

The SEM measurements of the hydrogels were recorded using the *VEGA3* SEM from TESCAN. This instrument is capable of recording structures of up to 20 kV and a magnification of 360,000. The hydrogels were freeze‐dried and sputter coated with 5 nm Platin. Images were taken of the cut surface and the surface at a magnification of 5,000.

#### 4.2.5 TGA

The TGA measurements of the textiles and extracted cellulose were carried out using a *TGA 2* instrument from Mettler Toledo. Nitrogen atmosphere was applied for analysis in the temperature range from 25 to 800°C. Around 100 mg of substance was analyzed in a 900 µL Al_2_O_3_ crucible.

All remaining TGA measurements were measured with the TGA 5500 from *TA Instrument*s. The measurements were carried out with platinum crucibles under a nitrogen atmosphere. The samples (5–10 mg) were heated with a heating rate of 10 K/min in a temperature range of 25–1000°C.

#### 4.2.6 Rheometer

The rheological measurements were carried out using an *ARES‐G2* rotational shear rheometer from TA Instruments. The resolution of the transducer torque is 1 nNm and the strain resolution is 0.04 µrad. Measurements were made with a strain amplitude of *γ*_0_ = 1% in the angular frequency range of 0.1–100 rad/s at 20°C.

#### 4.2.7 3D‐Printer

The BIONOVA‐X printer from Cellink is used to print the cellulose hydrogels. The wavelength of the LED lamp is 405 nm and the resolution is 10 µm. It is printed on an adhesive 24‐well plate. Printing was carried out at room temperature with a speed motion of 0.01 mm/s.

#### 4.2.8 Microscope

The *I* *mageXpress* Confocal HT.ai from Molecular Devices employs a seven‐channel laser light source with eight imaging channels, ensuring high throughput through the reduction of exposure times. This system is employed in the microscopic imaging of the scratch assay. The acquisition of these images is conducted over a duration of 48 h.

#### 4.2.9 Photometer

The *SpectraMax iD3* Multi‐Mode Microplate Reader from *Molecular Devices* was utilized to measure the absorbance of MTT 96 well plates. The measurement of these plates was conducted at a wavelength of 595 nm at room temperature.

## 5 Methods

### 5.1 Extraction of Cellulose From Textile Waste

Polycotton textile consisting of 50% polyester and 50% cotton received from Dibella BV as cutting remnants. A 100 mL pressure reactor from Büchi is used for the extraction. The reactor is heated to 40°C using a Julabo thermostat. The textile is cut into pieces with edge lengths of approximately 2.5 × 3 cm. 4 wt% of the textile in DMSO is used. 60 mL (66 g) of DMSO and 6.48 mL DBU are added to the reactor under a nitrogen atmosphere. The textile samples are placed in the solution and the reactor is flushed twice with 5 bar CO_2_. After 6 h of extraction at a pressure of 5 bar CO_2_ the stirrer is stopped, and the pressure is slowly released. The reactor content is filtered through a sieve with a mesh size of 1 mm. The textile pieces are stirred in 60 mL DMSO under 5 bar of CO_2_, for 30 min to rinse of the remaining cellulose and filtered off again. The cellulose solution is precipitated in 300 mL of ethanol. The residual textile is washed with 60 mL ethanol at least three times and dried at 80°C over night. The next day, the dried textile is extracted a second time using the same procedure. The precipitated cellulose from both extractions is combined and the ethanol of the precipitation bath is decanted of and switched to 600 mL of fresh ethanol. This is done four times in 72 h. Then, the cellulose is air–dried for several days and in a vacuum oven at 50°C at 100 mbar. After air drying, the composition of the residual textile and the extracted cellulose is determined using TGA.

### 5.2 Functionalization of Cellulose via Switchable Solvent System

Functional Cellulose was synthesized according to the literature [6]. Cellulose (1.50 mmol, 0.5 g, 1 eq.) was suspended in dry DMSO (10 mL) in a two neck round bottom flask before TBD (9.25 mmol, 1.28 g, 6.08 eq.) was added. The reaction mixture was stirred at 50°C in the presence of CO_2_ for 20 min until the solution became clear. The mixture was heated up to 95°C and methyl 10‐undecenoate (9.25 mmol, 2 mL, 6.08 eq.) was added dropwise. The mixture was stirred for 3 h under air flow to blow the methanol produced during the reaction out again through the open second neck. The crude product was diluted with 10 mL dry DMSO and precipitated in 200 mL deionized H_2_O. The residue was filtered and stirred in 200 mL of methanol for one night. Subsequently, after filtration, the product was dried under reduced pressure over night.

The entire reaction was repeated using recycled cellulose. The cellulose was first pulverized and then the reaction was carried out as previously described. After the addition of TBD and CO_2_, the solution was stirred at 50°C for 3–4 h instead of just 20 min with 30 mL DMSO instead of 10 mL until it dissolved. Subsequently, methyl 10‐undecenoate was added and the solution was stirred for 1 day under a nitrogen supply. The recycled cellulose was precipitated in H_2_O, washed in methanol overnight and then filtered off and dried under pressure.

For the simultaneous separation of polycotton and modification of cellulose extractions with 4 wt% polycotton (1.32 g) and 10 wt% DBU (3.24 mL) in 30 mL DMSO at 40°C and 5 bar CO_2_ carried out. After an extraction time of 6 h, the cellulose solution is filtered into a two neck round bottom flask using a sieve with a mesh size of 1 mm. The remaining textile is stirred in 30 mL fresh DMSO for 30 min under 5 bar CO_2_ and filtered into the flask as before. The flask is heated to 95°C and methyl 10‐undecenoate (24.37 mmol, 5.4 mL) is added with 6 eq. regarding the cellulose content of the polycotton is added dropwise under stirring. The flask is flushed with air while stirring to expel methanol for 16 h. The solution is added dropwise into a 500 mL ethanol bath for precipitation under vigorous stirring. The precipitated modified cellulose is filtered of and stirred in 500 mL fresh EtOH for at least 2 h. The washing is repeated three times.

### 5.3 Synthesis of PEG‐Dithiol

Dithiol‐functionalized PEG was synthesized according to the literature [10]. PEG 600 g/mol (8.33 mmol, 5 g, 1 eq.) and MPA (83.33 mmol, 8.84 g, 10 eq.) are dissolved in 30 mL toluene. The solution is heated to 50°C and *p*‐TSA (0.83 mmol, 0.16 g, 0.1 eq.) is added. The reaction mixture is heated to 130°C with stirring and is refluxed overnight. The mixture is then precipitated twice in 60 mL of cold diethyl ether and filtered off. After filtration it is dried overnight under reduced pressure. If there are still any acid residues in the product, the product is passed through an aluminum oxide column, which retains the acid and provides a pure product. The Table 3 below provides a list of masses for the various PEGs.

**TABLE 3:** Masses of the reactants for the synthesis of the various PEG dithiols.

	Mass of PEG, g	Mass of MPA, g	Mass of *p*‐TSA, g
PEG‐300‐dithiol	5	17.69	0.32
PEG‐600‐dithiol	5	8.84	0.16
PEG‐2000‐dithiol	5	2.64	0.048
PEG‐6000‐dithiol	5	0.88	0.016

### 5.4 Preparation of Cellulose‐Based Hydrogels

For 1 mL of 3 wt% hydrogel solution, modified cellulose (0.19 mmol, 30 mg, 1 eq.) is weighed into a photographic vial and dissolved in 500 µL of DMSO. PEG 600 g/mol (0.04 mmol, 25.2 mg, 1 eq.) with a ratio of 1:1.5 from double bond to thiol group is weighed into a snap glass and dissolved in 500 µL DMSO. The solutions are placed on the shaker until the solids are completely dissolved. The PEG‐dithiol solution is then added to the cellulose solution in the phototube and the photoinitiator LAP (0.0004 mmol, 0.12 mg, 0.01 eq.) is then added to the cellulose‐PEG solution. The vial is tightly sealed with a crimp closure and the solution is degassed with nitrogen. The resulting air bubbles are removed by centrifuging the solution at 700 rpm for 2 min. After removing all air bubbles, the vial is then placed 1 cm above a 3 Watt LED (Figure S13 in Supporting Information) with a wavelength of 405 nm and irradiated for 1 h at a power of 10 V and a current of 0.4 A.

### 5.5 3D‐Printing of Cellulose‐Based Hydrogels

3D‐printing is also used for gel formation. The DLP printer works using a light source that precisely crosslinks the polymer solution at predetermined points. A cube mold with nine small evenly spaced holes and a hollow cube are employed as the printing templates. The solution was prepared as previously described. As the light source of the DLP printer is not sufficiently intense, the LAP concentration was doubled. Following degassing, it was carefully poured into an adhesive 24‐well plate from *Cellink*, ensuring that no air bubbles were generated. The plate was then placed in the 3D printer and the template was selected. The printing was carried out at a speed motion of 0.01 mm/s, which results in a printing time of about 12 min.

### 5.6 Cytotoxicity Assay

All work is carried out under the sterile bench. The cells are first thawed and seeded into a cell culture flask covered with DMEM containing 10% FBS, 1% L‐alanyl‐L‐glutamine and 1% penicillin‐streptomycin. The cells are then incubated at 37°C in 5% CO_2_. For use, 20 mL of a cell concentration of 10^5^ cells/mL must first be prepared. First, the cell culture flask is examined under the microscope for impurities and contamination. If none are found, the cell culture medium is aspirated from under the safety bench and 10 mL of sterile PBS is added to the flask to remove the remaining cell culture medium. This wash solution is also aspirated. Then 2 mL of trypsin/EDTA is added to the cells and the bottle is incubated at 37°C for 4 min. At the end of the incubation period, the cells are dislodged from the bottom of the bottle by gently tapping. The dissolution of the cells can be observed under a microscope. Instead of adhering flat to the bottom, small round cells can be seen floating in the solution. 6 mL of cell culture medium is added to the bottle again under the safety bench to inactivate the trypsin. The cells are then transferred to a centrifuge tube and centrifuged at 1400 rpm for 6 min. After 6 min, the supernatant is discarded. Some liquid is retained to resuspend the cell pellet. Subsequently, 10 mL of fresh cell culture medium is added to the tube. The cells are enumerated using the Neubauer chamber to calculate the cell concentration of the existing cell suspension and to prepare a cell suspension with a cell concentration of 10^5^ cells/mL. The calculated volume of the cell suspension is then transferred to a tube and filled to a volume of 10 mL with cell culture medium. The MTT assay is performed to determine the cytotoxicity of the hydrogels. It is performed under sterile conditions to avoid contamination of the cell culture. All liquids utilized for the preparation or dilution of the solution must be sterile. The experiment was performed with fibroblasts to investigate possible toxicity on the skin. The cell suspension, which had been prepared in advance, was transferred to a plastic tube. Then, 100 µL of the cell suspension was seeded in a 96‐well plate using a multichannel pipette. The edge of the well plate is not covered. The plates are then incubated for 24 h at 37°C and 5% CO_2_. The hydrogels, prepared with PEG‐300, −600, −2000, and –6000 g/mol as crosslinker, were placed in cell culture medium at 37°C for 1, 3, and 7 days. Each 30 mg of the hydrogel was replenished with 1 mL of medium. The previous medium is then aspirated from the well plate, and 100 µL of the test solution is pipetted into the plate.

Two plates are prepared: one with the purchased modified cellulose and one with the recycled cellulose. In the control group, the previous medium is extracted and fresh medium is introduced. The treated cells are then incubated for 24 h at 37°C and 5% CO_2_. In the positive control, 5 µL of a 20% Triton X‐100 solution is added to each well and the plate is then incubated for 5 min at room temperature. Subsequently, the light is switched off and the MTT dye solution is transferred to a plastic tray. Using a multichannel pipette, 10 µL of the solution are added to each well, and the plate is then incubated for 2 h at 37°C and 5% CO_2_. Subsequently, the stop solution is transferred to a plastic tray and 100 µL is added to each well. The plate is then incubated overnight at 37°C and 5% CO_2_. The absorbances of the formazan are subsequently measured using an ELISA microplate reader at a wavelength of 595 nm. In order to determine cytotoxicity, the measured data must be averaged within the repeat assay and then related to each other. For this purpose, the positive control is defined as zero (0% viability) and subtracted from all values. The untreated negative control is regarded as the reference point for 100% viability. The remaining measured values are then arranged into a percentage ratio.

### 5.7 Scratch Assay

The scratch assay was performed according to the literature [35]. It is initiated with the preparation of a cell suspension. A volume of 200 µL of the cell suspension is transferred into an 8‐well chamber slide from *ibidi* and incubated for 24 h at 37°C and 5% CO_2_. Following this, a scratch is made in the middle of each well using a 200 µL pipette tip. The cells are thereby detached from the well plate. The detached cells are then rinsed with DPBS and aspirated. The cells are then stained with CMFDA, a fluorescent dye that labels cells green. The cell medium in the cell culture dish is then aspirated, and the cells are covered with the staining solution. These are then incubated for a period of 1 h at a temperature of 37°C and a concentration of 5% CO_2_. The hydrogels are then placed on the cells. Given that the hydrogels are not prepared under sterile conditions, they must undergo a pretreatment before coming into contact with the cells. A stock solution of 10,000 units/mL penicillin and 10 mg/mL streptomycin is prepared in a 0.9% sodium chloride solution. The hydrogels in the wells are then coated with this solution and exposed to UV light overnight to achieve sterilization. Purchased and recycled cellulose hydrogels, composed of PEG‐2000 g/mol, are likewise prepared and subsequently placed on the cells poststerilization. Two wells are covered with the hydrogels containing the recycled cellulose, two with the purchased cellulose, and four without hydrogels, serving as a growth control. Microscopic images are obtained at specific time points over a 48 h period.

## Supporting Information

Additional supporting information can be found online in the Supporting Information section. **Supporting** **Fig.** **S1:** ^31^P NMR of MCC after functionalization phosphitylated in CDCl_3_/pyridine. **Supporting Fig. S** **2:** TGA measurements of virgin polycotton, residual textile and extracted cellulose. **Supporting Fig. S** **3:** ATR‐FTIR spectrum of recycled cellulose and recycled cellulose esters with methyl 10‐undecenoate after separation of polycotton and functionalization in one process. **Supporting Fig. S** **4:** ^1^H NMR of MCC and recycled cellulose after functionalization dissolved in DMSO‐d_6_. ^1^H NMR (400 MHz, DMSO‐d_6_) δH ppm: 5.83–5.76 (m, COCH_2_CH_2_CH_2_(CH)_4_CH_2_CH_2_‐CH=CH2), 5.02–4.92 (q, COCH_2_CH_2_CH_2_(CH)_4_‐CH_2_CH_2_CH=CH_2_), 2.34–2.29 (t, COCH_2_CH_2_CH_2_(CH)_4_CH_2_CH_2_CH=CH_2_), 2.02–1.99 (d, COCH_2_CH_2_CH_2_(CH)_4_CH_2_‐CH_2_CH=CH_2_), 1.52 (s, COCH_2_CH_2_CH_2_(CH)_4_‐CH_2_CH_2_CH=CH_2_), 1.34 (s, COCH_2_‐CH_2_CH_2_(CH)_4_CH_2_CH_2_CH=CH_2_), 1.26 (s, CO‐CH_2_CH_2_CH_2_(CH)_4_CH_2_CH_2_CH=CH_2_). **Supporting Fig. S** **5:** ATR‐FTIR of MCC and recycled cellulose after functionalization. ATR‐FTIR cm−1: 2928–2852 ν(C–H), 1738 ν(C=O), 1640 ν(C=C). **Supporting Fig. S** **6:** SEC measurements of MCC and recycled cellulose after functionalization in DMAc. The molecular weight evaluation was done by calibration with pullulan standards. SEC (DMAc, Pullulan standard): commercial cellulose: M_*n*_ = 108,000 g/mol, M_*w*_ = 800,000 g/mol, *Ð* = 7.36. recycled cellulose: M_*n*_ = 187,000 g/mol, M_*w*_ = 1,386,000 g/mol, *Ð* = 7.41. **Supporting Fig. S** **7:** 1H NMR spectrum of thiol‐functionalized PEG‐300‐, ‐600‐, ‐2000‐, ‐6000‐dithiol dissolved in CDCl3. 1H NMR (400 MHz, CDCl3) δH ppm: 4.18–4.15 (t, CH2CH2OCOCH2CH2SH ), 3.62–3.60 (t, CH2CH2‐OCOCH2CH2SH), 3.55 (s, CH2CH2CH2CH2), 2.70–2‐65 (q, CH2CH2SH), 2.60–2‐57 (t, CH2CH2SH), 1.62–1.58 (t, CH2CH2SH). **Supporting Fig. S** **8:** ATR‐FTIR spectrum of PEG‐300‐, ‐600‐, ‐2000‐, ‐6000‐dithiol. ATR‐FTIR cm−1: 2874 ν(C–H), 1731 ν(C=O), 1102 v(C‐O). **Supporting Fig. S** **9:** FTIR spectrum of hydrogels made from commercial and recycled cellulose with PEG‐300‐, ‐600‐, ‐2000‐ and ‐6000‐dithiols. **Supporting Fig. S** **10:** Swelling behavior of hydrogels made from commercial and recycled cellulose with PEG‐300‐, ‐600‐, ‐2000‐ and ‐6000‐dithiols. **Supporting Fig. S** **11:** TGA measurements of swollen hydrogels made from commercial and recycled cellulose with PEG‐300‐, ‐600‐, ‐2000‐ and ‐6000‐dithiols. **Supporting Fig. S** **12:** TGA measurements of freeze‐dried hydrogels made from commercial and recycled cellulose with PEG‐300‐, ‐600‐, ‐2000‐ and ‐6000‐dithiols. **Supporting Fig. S** **13:** Setup of the crimp vials 1 cm above 405 nm LEDs for gel formation. **Supporting Fig. S** **14:** 3D‐printed cubes of cellulose from a 3 wt% solution with 1:1.5 crosslinker ratio and 1 mg/mL LAP in DMSO after 3 months storage.

## Funding

This study was supported by Helmholtz‐Gemeinschaft (43.33.11).

## Conflicts of Interest

The authors declare no conflicts of interest.

## Supporting information

Supplementary Material

## Data Availability

The data that support the findings of this study are openly available in RADAR4CHEM at https://doi.org/10.22000/n8gp2quef7zz5vcf, reference number 1.
