# Supplementary material for: 3D‐Printed Hydrogels from Recycled Cellulose for Biomedical Applications
Source: ChemSusChem. 2025 Dec 29;19(1):e202501734. doi: 10.1002/cssc.202501734 (PMC12746703; doi:10.1002/cssc.202501734)
Supplement: Supplementary file 1 — Supplementary Material [file CSSC-19-e202501734-s001.pdf]

3D-Printed Hydrogels from Recycled Cellulose for Biomedical Applications

Supporting Information

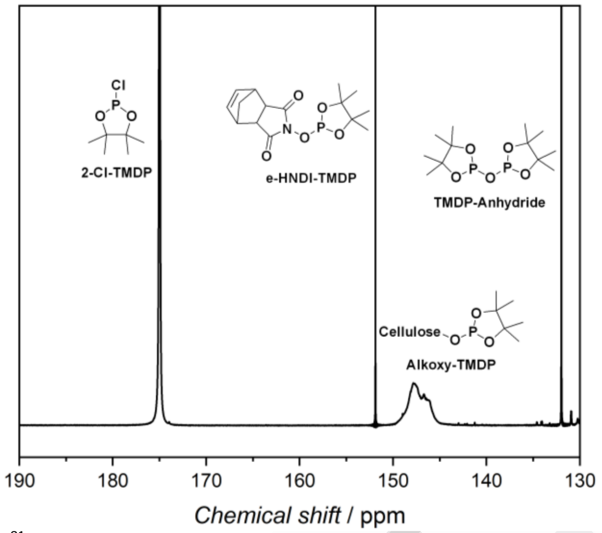

Figure S1. ^{31}P NMR of MCC after functionalization phosphitylated in $CDCl_3$/pyridine.

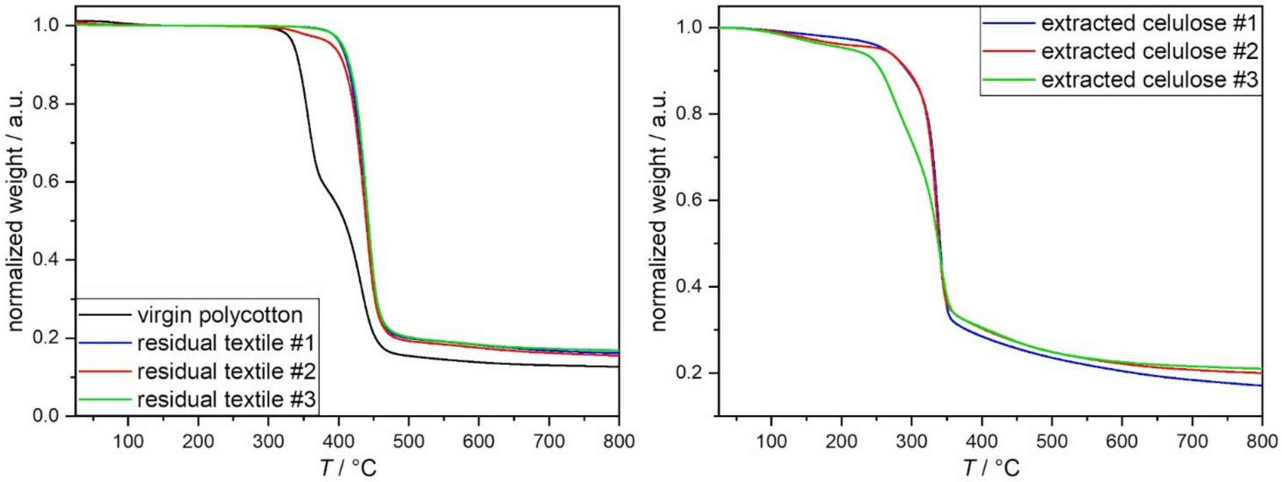

Figure S2. TGA measurements of virgin polycotton, residual textile and extracted cellulose.

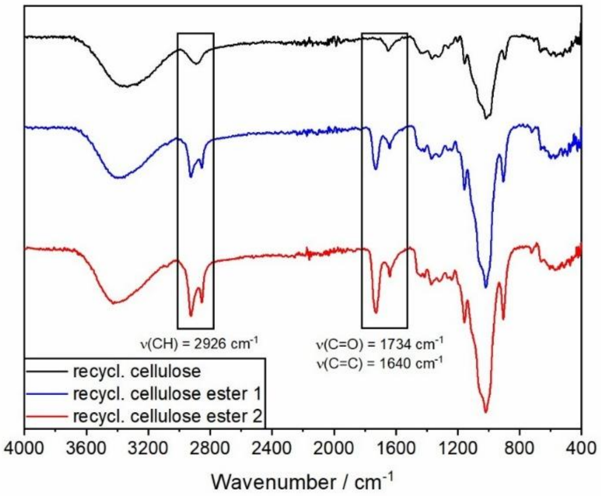

Figure S3. ATR-FTIR spectrum of recycled cellulose and recycled cellulose esters with methyl 10-undecenoate after separation of polycotton and functionalization in one process.

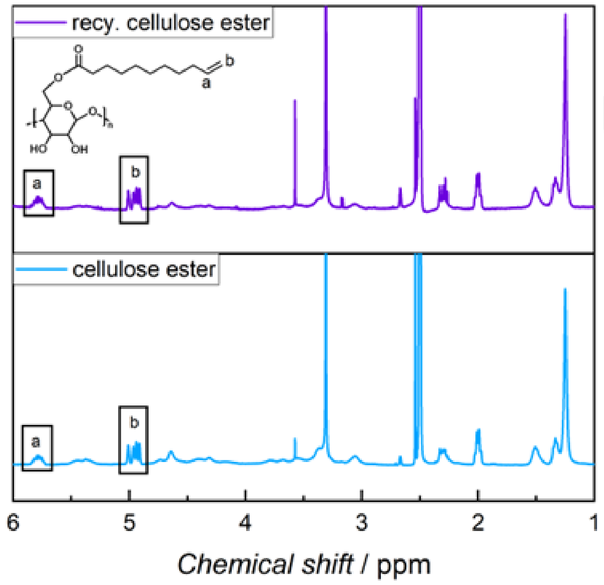

Figure S4. ^{1}H NMR of MCC and recycled cellulose after functionalization dissolved in DMSO-d_6. ^{1}H NMR (400 MHz, DMSO-d_6) δH ppm: 5.83–5.76 (m, $COCH_2CH_2CH_2(CH)_4CH_2CH_2$-$CH=CH_2$), 5.02–4.92 (q, $COCH_2CH_2CH_2(CH)_4$-$CH_2CH_2CH=CH_2$), 2.34–2.29 (t, $COCH_2CH_2CH_2(CH)_4CH_2CH_2CH=CH_2$), 2.02–1.99 (d, $COCH_2CH_2CH_2(CH)_4CH_2$-$CH_2CH=CH_2$), 1.52 (s, $COCH_2CH_2CH_2(CH)_4$-$CH_2CH_2CH=CH_2$), 1.34 (s, $COCH_2$-$CH_2CH_2(CH)_4CH_2CH_2CH=CH_2$), 1.26 (s, CO-$CH_2CH_2CH_2(CH)_4CH_2CH_2CH=CH_2$).

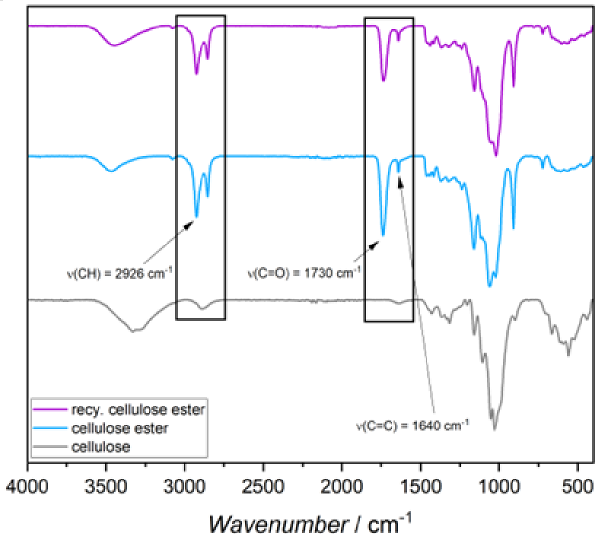

Figure S5. ATR-FTIR of MCC and recycled cellulose after functionalization. ATR-FTIR cm^{-1}: 2928–2852 ν(C–H), 1738 ν(C=O), 1640 ν(C=C).

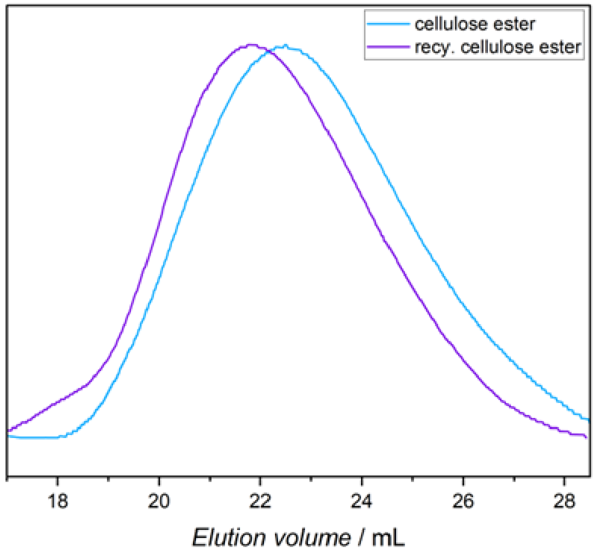

Figure S6. SEC measurements of MCC and recycled cellulose after functionalization in DMAc. The molecular weight evaluation was done by calibration with pullulan standards. SEC (DMAc, Pullulan standard): commercial cellulose: M_n = 108,000 g/mol, M_w = 800,000 g/mol, $Đ$ = 7.36. recycled cellulose: M_n = 187,000 g/mol, M_w = 1,386,000 g/mol, $Đ$ = 7.41.

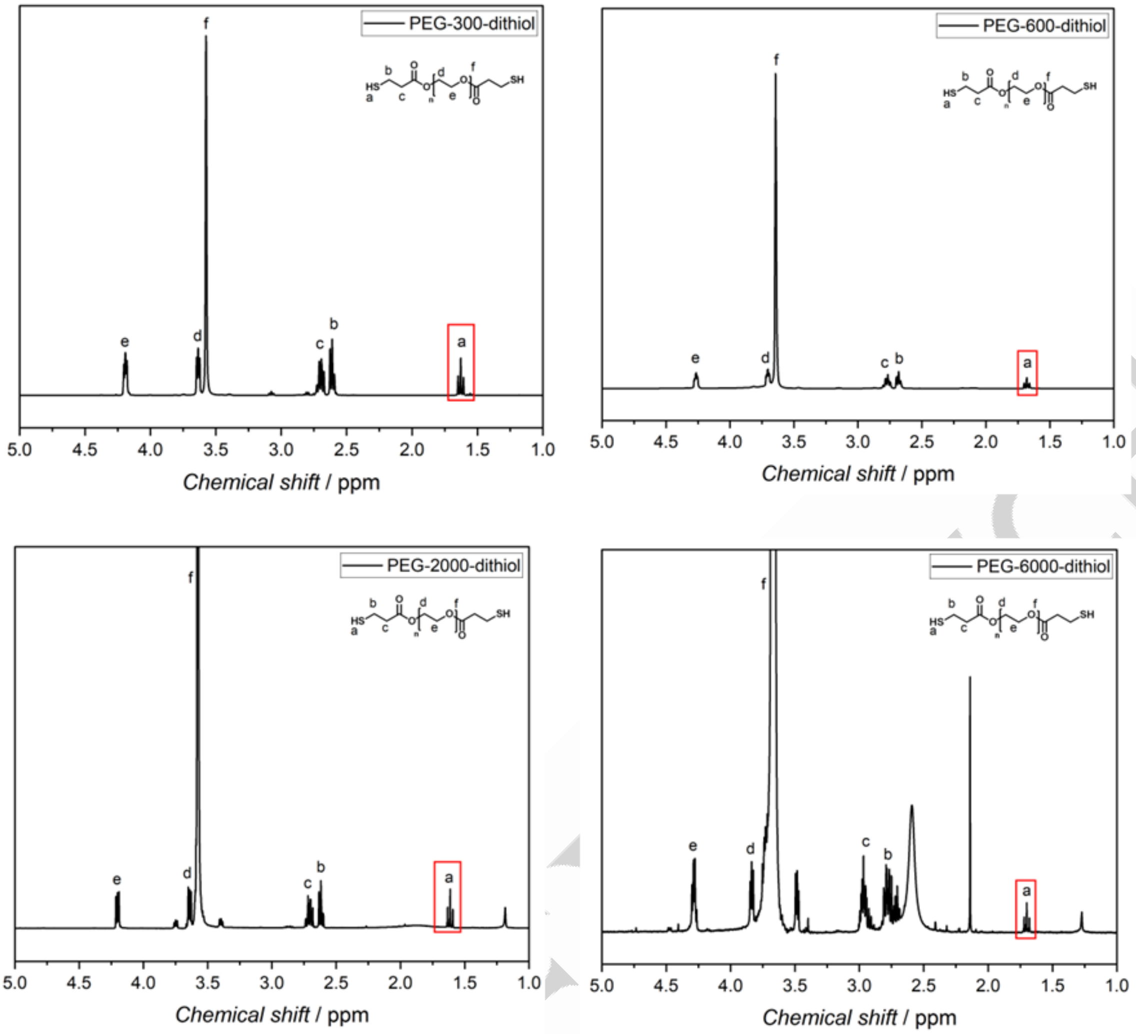

Figure S7. ^{1}H NMR spectrum of thiol-functionalized PEG-300-, -600-, -2000-, -6000-dithiol dissolved in $CDCl_3$. ^{1}H NMR (400 MHz, $CDCl_3$) δH ppm: 4.18–4.15 (t, $CH_2CH_2OCOCH_2CH_2SH$), 3.62–3.60 (t, CH_2CH_2-$OCOCH_2CH_2SH$), 3.55 (s, $CH_2CH_2CH_2CH_2$), 2.70–2-65 (q, CH_2CH_2SH), 2.60–2-57 (t, CH_2CH_2SH), 1.62–1.58 (t, CH_2CH_2SH).

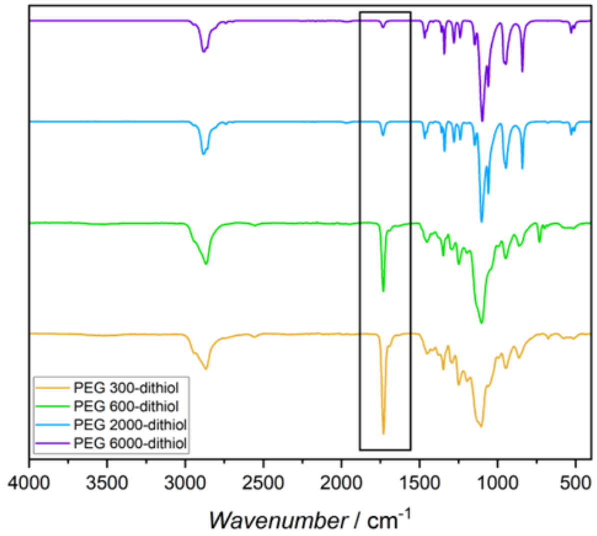

Figure S8. ATR-FTIR spectrum of PEG-300-, -600-, -2000-, -6000-dithiol. ATR-FTIR cm^{-1}: 2874 ν(C–H), 1731 ν(C=O), 1102 ν(C-O).

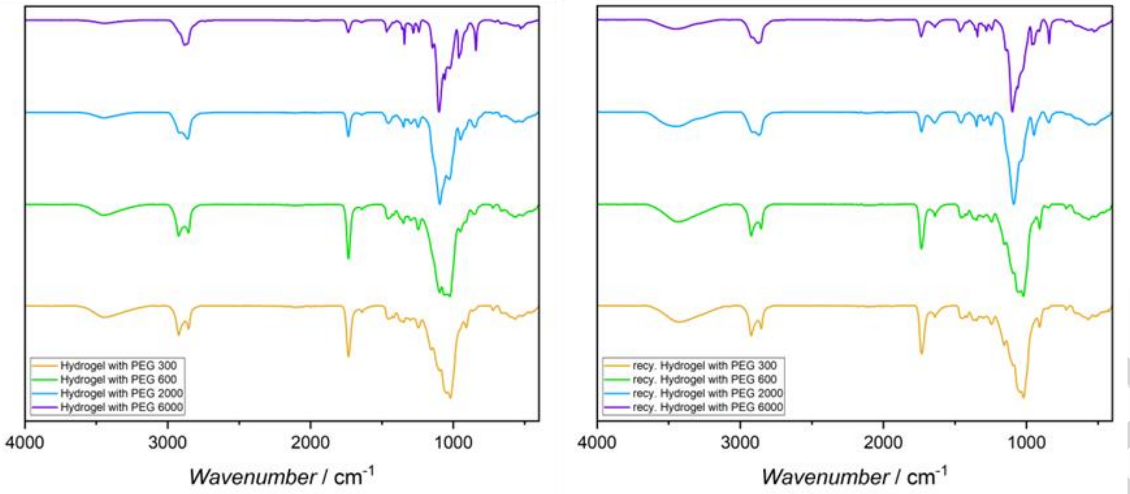

Figure S9. FTIR spectrum of hydrogels made from commercial and recycled cellulose with PEG-300-, -600-, -2000- and -6000-dithiols.

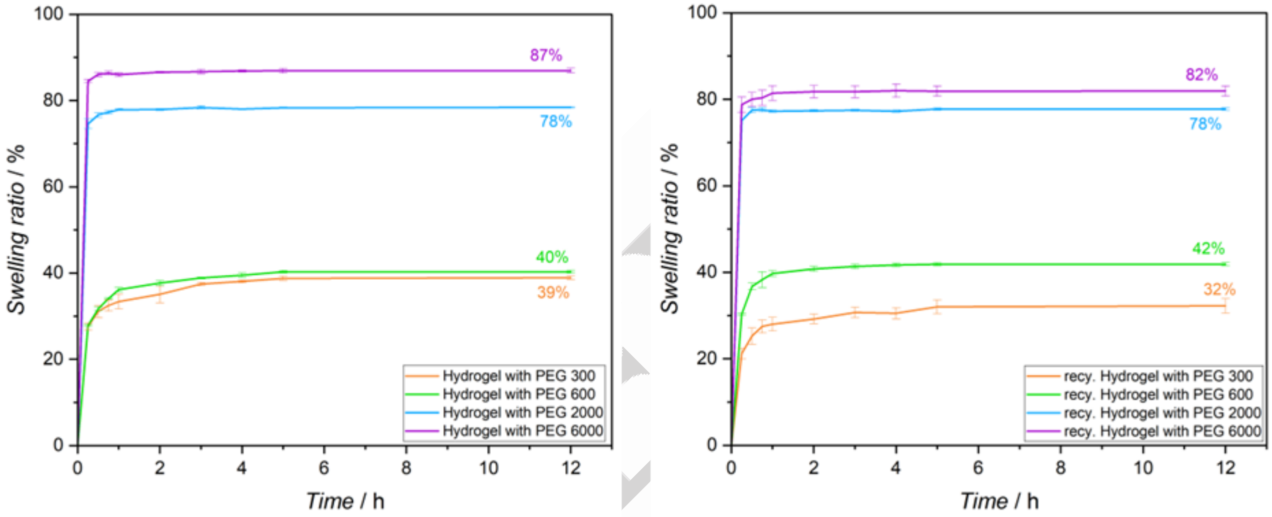

Figure S10. Swelling behavior of hydrogels made from commercial and recycled cellulose with PEG-300-, -600-, -2000- and -6000-dithiols.

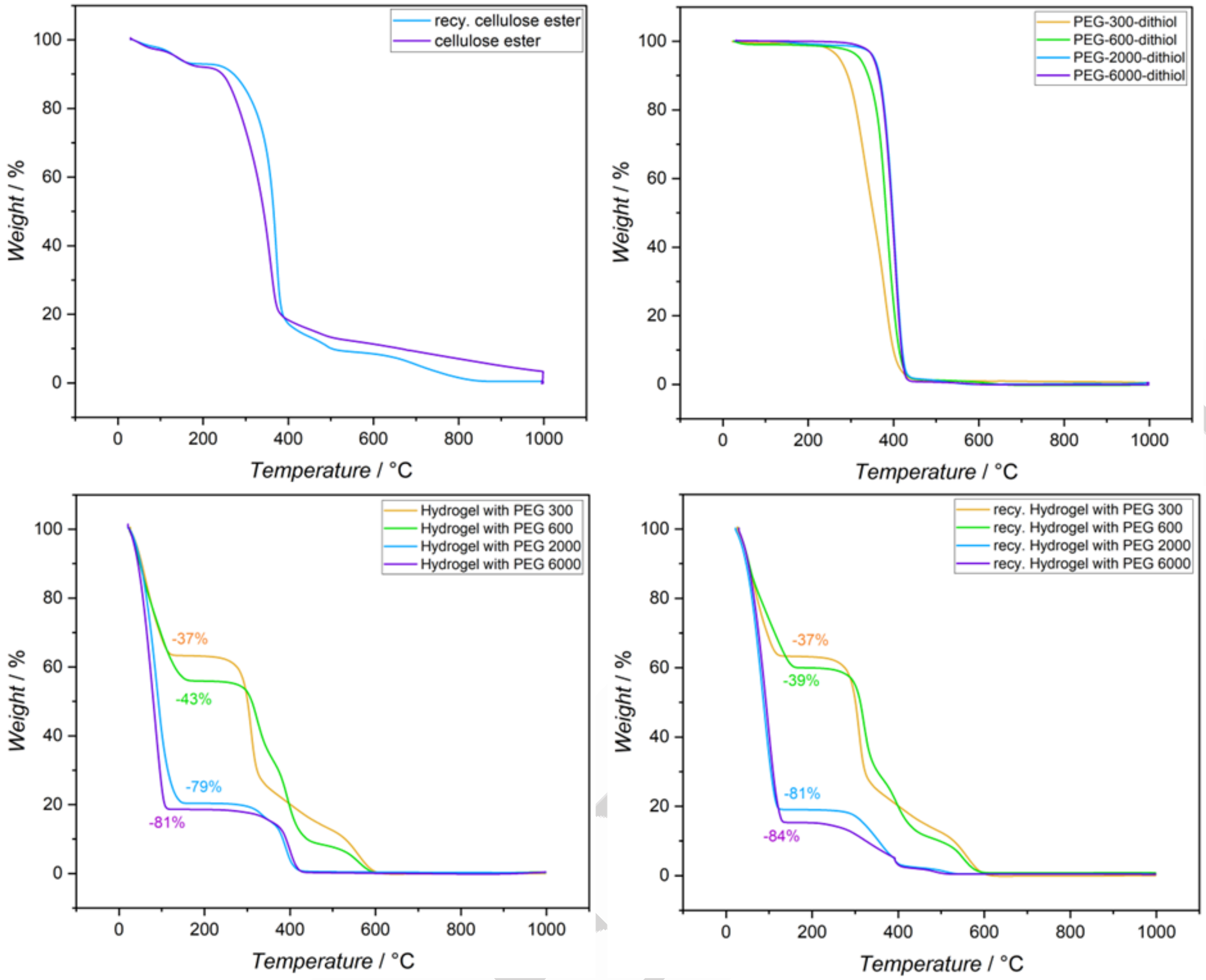

Figure S11. TGA measurements of swollen hydrogels made from commercial and recycled cellulose with PEG-300-, -600-, -2000- and -6000-dithiols.

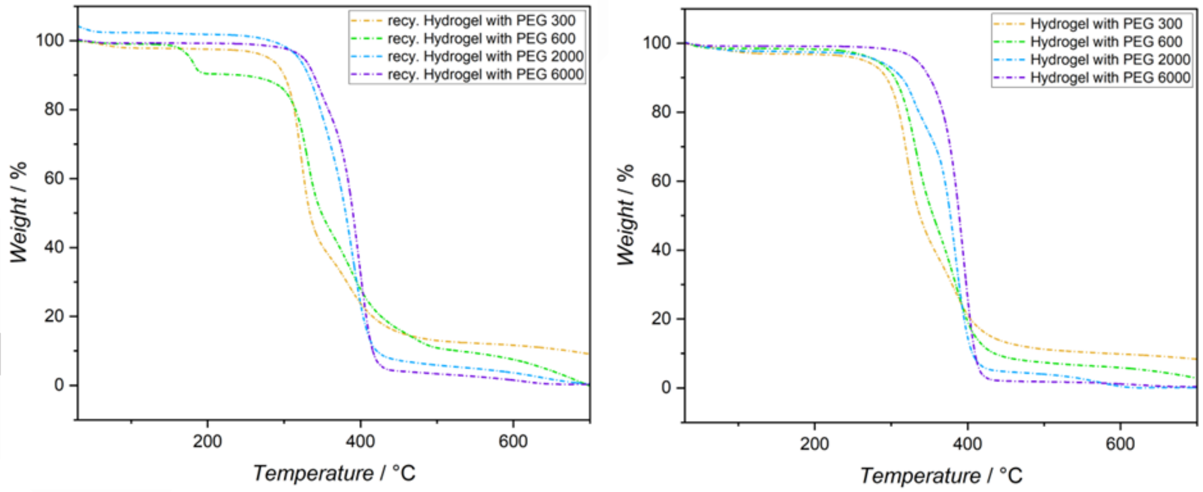

Figure S12. TGA measurements of freeze-dried hydrogels made from commercial and recycled cellulose with PEG-300-, -600-, -2000- and -6000-dithiols.

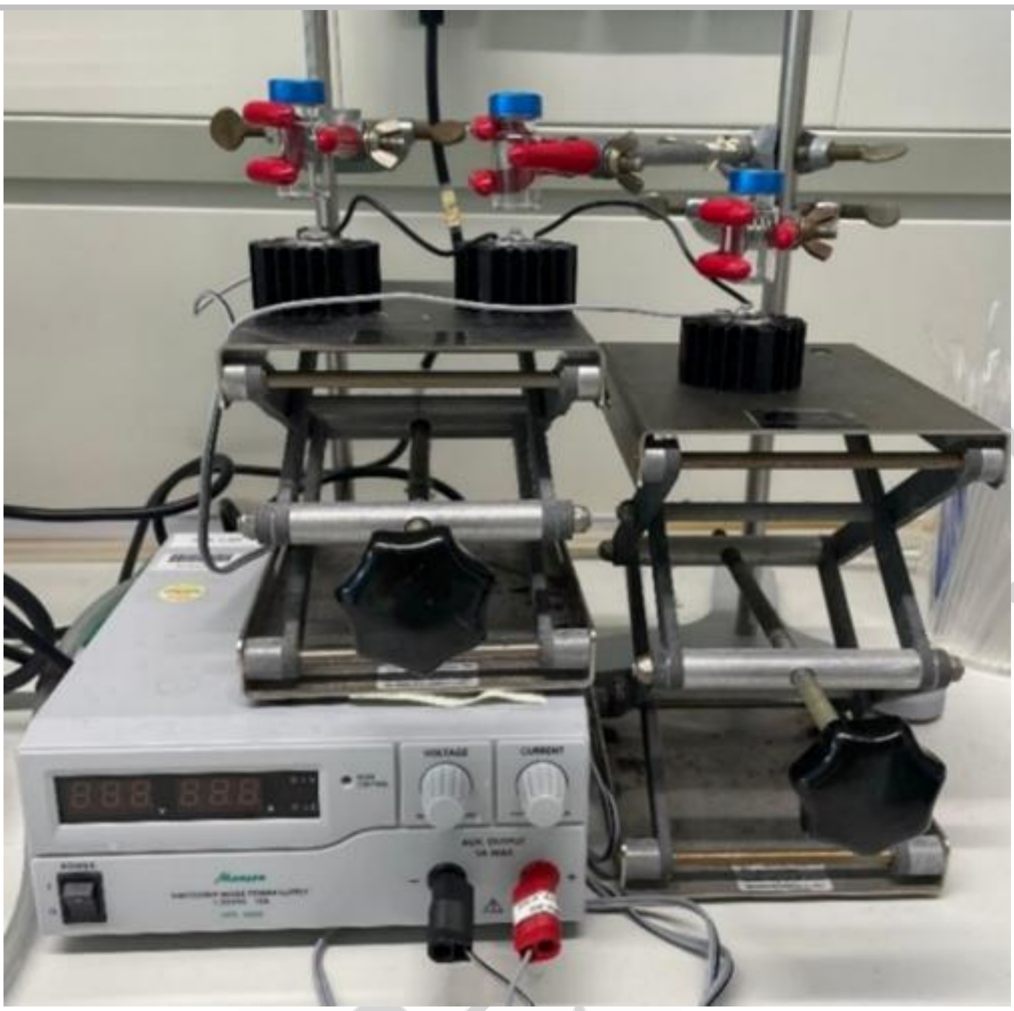

Figure S13. Setup of the crimp vials 1 cm above 405 nm LEDs for gel formation.

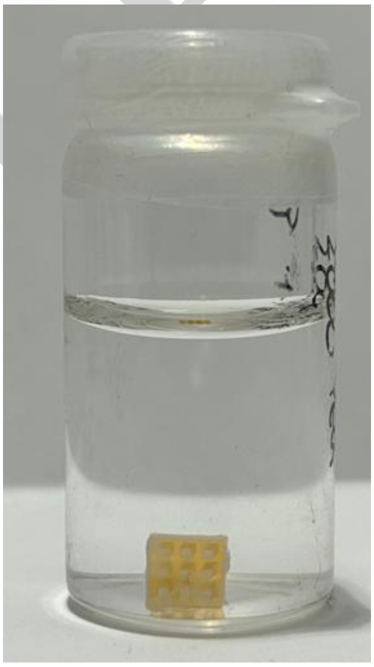

Figure S14. 3D-printed cubes of cellulose from a 3 wt% solution with 1:1.5 crosslinker ratio and 1 mg/mL LAP in DMSO after 3 months storage.
